# The role of size constancy for the integration of local elements into a global shape

**DOI:** 10.3389/fnhum.2013.00342

**Published:** 2013-07-03

**Authors:** Johannes Rennig, Hans-Otto Karnath, Elisabeth Huberle

**Affiliations:** ^1^Division of Neuropsychology, Center of Neurology, Hertie-Institute for Clinical Brain Research, University of TübingenTübingen, Germany; ^2^Department of Psychology, University of South CarolinaColumbia, SC, USA; ^3^Neurology and Neurorehabilitation Center, Luzerner KantonsspitalLuzern, Switzerland

**Keywords:** visual constancy, global perception, Gestalt, Kanizsa, visual context, perceptual grouping

## Abstract

Visual perception depends on the visual context and is likely to be influenced by size constancy, which predicts a size and distance invariant perception of objects. However, size constancy can also result in optical illusions that allow the manipulation of the perceived size. We thus asked whether the integration of local elements into a global object can be influenced by manipulations of the visual context and size constancy? A set of stimuli was applied in healthy individuals that took advantage of the “Kanizsa” illusion, in which three circles with open wedges oriented toward a center point are placed to form an illusionary perception of a triangle. In addition, a 3D-perspective view was implemented in which the global target (“Kanizsa” triangle) was placed in combination with several distractor circles either in a close or a distant position. Subjects were engaged in a global recognition task on the location of the “Kanizsa” triangle. Global recognition of “Kanizsa” triangles improved with a decreasing length of the illusory contour. Interestingly, recognition of “Kanizsa” triangles decreased when they were perceived as if they were located further away. We conclude that the integration of local elements into a global object is dependent on the visual context and dominated by size constancy.

## Introduction

Principles of Gestalt perception have strongly influenced our understanding of visual cognition. In the past century, Gestalt psychologists like Koffka (1935) and Wertheimer (1923) postulated that the human brain determines single elements with common features as a single entity rather than a sum of separate parts. Stimuli used to investigate the integration of multiple elements into a complex object can be found in Navon letters (Navon, 1977), i.e., hierarchically organized visual stimuli where a global letter is constructed from an array of local letters, or the “Kanizsa” illusion (Kanizsa, 1955). This illusion consists of an arrangement of circles with a wedge like gap (so called “pacman”) that form an illusory angular figure. The “Kanizsa” illusion evokes neuronal responses similar to “real” geometrical figures in early and higher visual areas along the ventral stream (Hirsch et al., 1995; Ffytche and Zeki, 1996; Larsson et al., 1999; Halgren et al., 2003; Stanley and Rubin, 2003; Maertens and Pollmann, 2005, 2007; Maertens et al., 2008). Regarding the spatial characteristics of “Kanizsa” illusions it has been demonstrated that their global recognition performance decreases with an increasing length of the illusory contours (Kojo et al., 1993; Liinasuo et al., 1997). This observation is in line with a recent study showing that the spatial distance between local letters is crucial for recognition of Navon letters in neurological patients with simultanagnosia (Huberle and Karnath, 2006).

Besides physical properties of objects themselves, the visual environment plays an important role for efficient object recognition. In this context, the phenomenon of visual constancy is a crucial factor of human visual perception. Visual constancy is a key mechanism that allows the perception of familiar objects at a “standardized” shape, size, or color and is also critical for the invariant identification of objects regardless of changes in perspective, distance, lighting or the size of the retinal image (Emmert, 1881; Brunswik, 1934; Hebb, 1958; Fitzpatrick et al., 1982). Various perceptual illusions like the Ponzo or the Müller-Lyer illusion (Müller-Lyer, 1889; Ponzo, 1911) are explained by size constancy—a crucial aspect of visual constancy enabling invariant size perception. Moore and Egeth (1997) revealed a pre-attentional influence for grouping mechanisms in the way that the length estimation of simple lines presented within a dot array was affected by the configuration of the dots in the background. When these dots formed a Ponzo or Müller-Lyer illusion, the length estimation changed depending on the arrangement of the surrounding dots. Importantly, the effect was present although the participants were unaware of the dot configurations in the background.

A previous study (Beck, 1975) demonstrated a significant role of perspective on global recognition performance. Global perception improved if the global target was perceived further away by tilting the stimulus; global recognition was supported by this perspective change which produced a closer retinal spacing between local elements. We therefore asked whether global recognition performance is dependent on the perceived distance between the individual elements of hierarchically organized stimuli and hypothesized that a perspective manipulation inducing larger distances between local elements by means of size constancy might result in decreased global recognition. In contrast to the work by Beck (1975), the present study aimed to achieve a distance manipulation by mechanisms of size constancy preserving a constant retinal image. In detail, we presented healthy observers with a 3D perspective view of an edged wall, in which an illusory “Kanizsa” triangle in an array of distractors was placed either at the close (Front condition) or the distant (Back condition) part of the wall. We assumed that the distant section of the wall would be perceived subjectively larger compared to the close section although the physical and retinal image remained unchanged. If size constancy influences Gestalt perception, the illusory contours of the “Kanizsa” triangles presented in the Back condition should be more difficult to perceive (cf. Beck, 1975), resulting in a decrease in recognition performance.

## Methods

### Participants

Twenty healthy individuals (5 males, 15 females; average age 24.0 years, *SD* = 3.9) participated in Experiments 1 and 2; another 20 observers (6 males, 14 females; average age 25.5 years, *SD* = 4.4) took part in Experiments 3 and 4; 22 subjects (2 males, 20 females; average age 23.4 years, *SD* = 4.1) were tested in Experiment 5. All participants had normal or corrected-to-normal vision, no history of brain damage, and gave their informed consent before the participation in the study, which has been performed in accordance with the ethical standards laid down in the 1964 Declaration of Helsinki.

In Experiments 2 and 4, one participant had to be excluded for the final analysis due to low performance. Even in the easiest stimulus conditions where all other subjects showed an accuracy between 80–100%, this subject's performance did not exceed the 50% chance rate. We attributed this behavior to a lack of motivation or comprehension. In Experiment 5, three participants had to be excluded due to the same reason.

### Stimuli

#### Experiment 1

Stimuli displayed an edged stone wall with a perpendicular arrangement of the different parts of the wall (Figure 2A). The two main parts of the wall were oriented in parallel to the horizontal outline of the stimuli and appeared to be located close to the observer (Front) or further away (Back). In addition, the Front could be located to the left or the right from the center of the stimulus. All stimuli had a size of 19° × 19° visual angle, in which the Front covered an area of 19.0° × 9.5° with a size of the individual stones of 3.5° × 1.0°. The size of the Back was 7.0° × 5.0° with a size of individual stones of 1.4° × 0.4°. A fixation dot was placed at the center of the stimulus.

**Figure 1:**
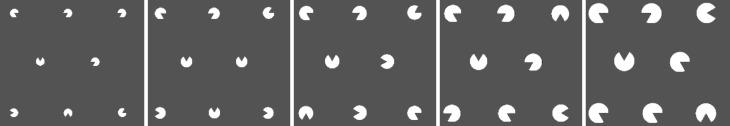
**Five 3-2-3 arrays with the five sizes of pacmen, with a Kanizsa triangle in the left upper corner; from left to right: Size 1–5**.

Finally, a 3-2-3 array of white circles composed of wedge-like gaps (also known as “pacman”) was superimposed on the wall. The size of the array was 4.0° × 4.0°. The center of this array was placed in the center of the Front or the Back and thus located 6.5° to the right or the left from the central fixation dot. Three of the eight “pacmen” were oriented to enable the perception of an illusory triangle, while the remaining “pacmen” had a random orientation. The triangle was located at one of following positions within the array: (1) top/left, (2) top/right, (3) bottom/left, (4) bottom/right. The four possible positions were balanced for both presentation conditions (Front, Back) and sides (left, right) in all experiments. In order to facilitate the integration of the local “pacman” into a global triangle, we manipulated the size of the “pacmen.” In detail, the following sizes of the “pacmen” were used: 0.4° (Size 1), 0.5° (Size 2), 0.6° (Size 3), 0.7° (Size 4), and 0.8° (Size 5). The distance of 1.8° (calculated from the center of the “pacmen”) between the individual elements remained unchanged across conditions. Examples of the five sizes are shown in Figure 1.

#### Experiment 2

**Figure 2:**
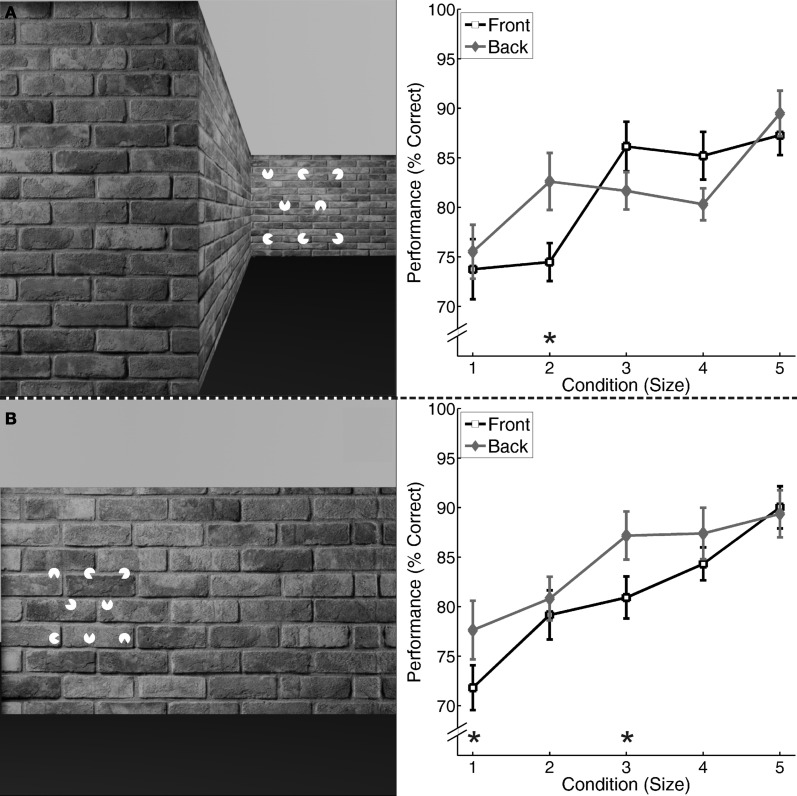
**Left: 3-2-3 array of white “pacmen” forming a “Kanizsa” triangle in one of four possible positions presented on one side of a shifted wall. (A)** Stimulus for Experiment 1: 3-2-3 array presented on a naturalistic wall; **(B)** Stimulus for Experiment 2: 3-2-3 array presented on a wall appearing close (Front condition). The distant condition (Back) is not shown explicitly. **Right:** Results of Experiments 1 and 2 **(A,B)**: displayed is the average percentage and standard errors of correct identification of “Kanizsa” triangles for the five different conditions of the “pacman” (Size 1–5) and two locations (Front, Back). ^*^p < 0.01, Bonferroni corrected for five comparisons.

Experiment 2 aimed to test for differences in the integration of local elements into a global shape irrespective to the global perspective, but with respect to the local surround. The stimuli displayed a straight wall without the presence of edges (Figure 2B). The vertical extension of this wall was 11.0°, which was the average of the Front and Back in Experiment 1. In parallel to Experiment 1, the individual stones covered a size of 1.4° × 0.4° (Back) or 3.5° × 1.0° (Front). The stimulus parameters of the 3-2-3 array were identical to Experiment 1.

#### Experiment 3

**Figure 3:**
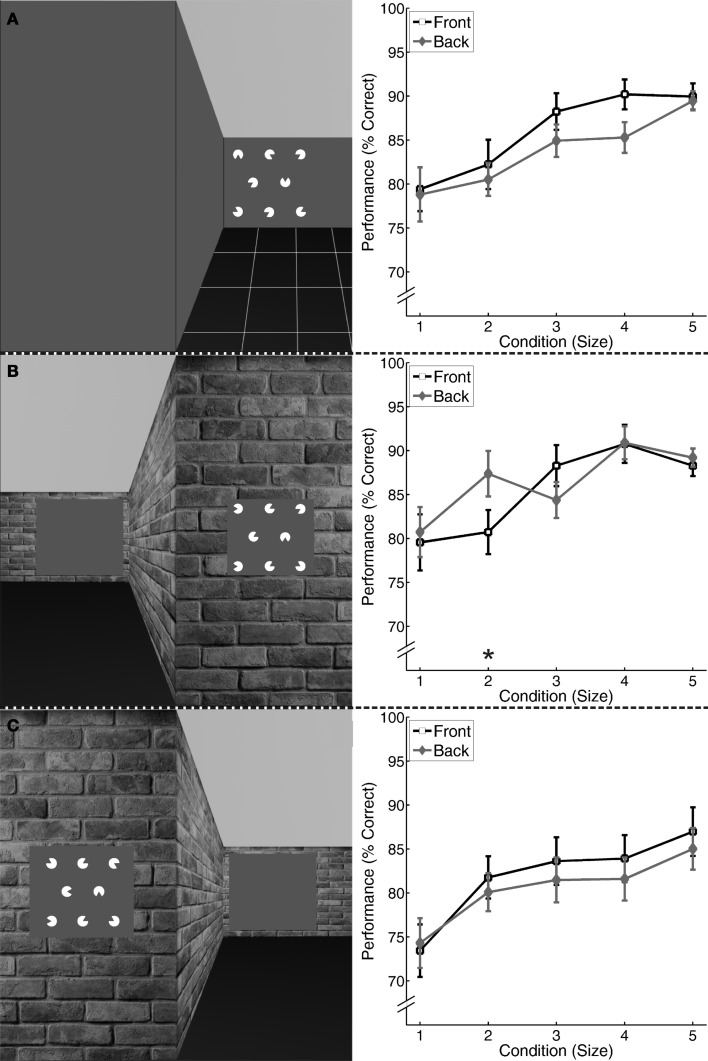
**Left: 3-2-3 array of white “pacmen” forming a “Kanizsa” triangle in one of four possible positions presented on one side of a shifted wall. (A)** Stimulus for Experiment 3: 3-2-3 array presented on a uniform gray wall; **(B)** Stimulus for Experiment 4: 3-2-3 array presented on a gray box within a naturalistic wall. **(C)** Sample Stimulus for Experiment 5: 3-2-3 array presented on a size adjusted gray box within a naturalistic wall (6.4° × 7.0°, which was the size closest to the mean perceived size). **Right:** Results of Experiments 3–5 **(A–C)**: displayed is the average percentage and standard errors of correct identification of “Kanizsa” triangles for the five different conditions of the “pacman” (Size 1–5) and two locations (Front, Back). ^*^p < 0.01, Bonferroni corrected for five comparisons.

Experiment 3 aimed to test for differences in the integration of local elements into a global shape irrespective of the local surround, but with respect to the global perspective. The stimuli for Experiment 3 were comparable to the stimuli used for Experiment 1 with the exception that individual stones were replaced by a uniform gray surface (average of all pixels belonging to the stone wall in Experiment 1; Figure 3A). The 3D-perspective was generated by a squared pattern on the bottom in front of the wall.

#### Experiment 4

In parallel to Experiment 3, Experiment 4 aimed to test for differences in the integration of local elements into a global shape irrespective of the local surround, but with respect to the global perspective. The 3-2-3 array was embedded in a gray square (average of all pixels belonging to the stone wall in Experiment 1; Figure 3B) with a size of 4.8° × 5.4°, while the remaining stimulus parameters were identical to Experiment 1.

#### Experiment 5

Experiment 5 should control the possibility of an influence of local perspective effects by the size of the gray square used in Experiment 4. It consisted of two different parts: a preliminary test and the main experiment. In the preliminary test, the stimulus set of Experiment 4 was used but the 3-2-3 array was removed and a second gray square was added. In addition, the size of the gray square in the Back remained constant (4.8° × 5.4°) while the size of the square in the Front was presented in ten different sizes (4.8° × 5.4°, 5.0° × 5.6°, 5.2° × 5.8°, 5.4° × 6.0°, 5.6° × 6.2°, 5.8° × 6.4°, 6.0° × 6.6°, 6.2° × 6.8°, 6.4° × 7.0°, 6.6° × 7.2°). Figure 3C shows a square of 6.4° × 7.0°, which was the size closest to the mean perceived size (see Results). We then determined the condition, in which the participant's perceived an equal size of the two gray squares. This condition was used for the main experiment, which was identical to Experiment 4.

### Procedure

All experiments were conducted in a room with dimmed light; stimuli were presented on a 19 inches CRT monitor with subjects located 57 cm in front of it. Stimulus presentation and data collection were controlled by a custom-made program using MatLab 2003b (MathWorks) and the Psychophysics Toolbox (Version 2.54; Brainard, 1997).

#### Experiments 1–4

The same design and procedure were used for Experiments 1–4. Each experiment consisted of ten conditions (Front: Size 1–5, Back: Size 1–5) that were repeated 48 times, resulting in a total number of 480 trials. All trials were presented in a random order in three blocks of 160 trials each. Before the onset of the first trial, all observers were familiarized with the type of stimuli and task in a short practice session.

Each trial started with a stimulus presentation of 250 ms followed by a blank interval between 2250 and 3250 ms, in which the central fixation dot was presented in a gray background. This procedure resulted in a trial duration between 2500 and 3500 ms. During the blank interval, participants were engaged in a two alternative forced choice task on the position of the triangle. That is, they were instructed to indicate, if the location of the perceived triangle was at the top or the bottom of the 3-2-3 array by pressing a button in their right or left hand. The design was balanced for the position of the target triangle as well as the location of the array (left or right) with respect to its position (Front or Back). Further, the keymapping was counterbalanced across participants. Participants were instructed to maintain fixation throughout the study.

#### Experiment 5

The preliminary test consisted of ten conditions (Size 1–10) that were repeated 16 times each resulting in a total number of 160 trials. All trails were presented within one block. The participants were instructed to indicate which of the two squares was perceived larger. The condition of subjective equality was used for the main part of Experiment 5, which was identical to Experiments 1–4.

### Results

#### Experiment 1

Figure 2A shows that the percentage of correct responses tended to increase with an increasing size of the “pacman” for both Front and Back presentation conditions. We performed a repeated-measures ANOVA on the percentage of correct responses with Size (Size 1–5) and Position (Front, Back) as independent factors. The analysis revealed a significant interaction between the two factors [*F*_(4, 16)_ = 4.50, *p* = 0.01]. Simple main effects were investigated by comparing Front and Back positions for every size of the “pacman” by paired *T*-tests. Using a Bonferroni-correction to correct for multiple comparisons (resulting in an alpha level of.01) we observed significant differences for Size 2 [2: *T*_(19)_ = 3.63, *p* = 0.002], but not for Sizes 1 and 3–5 [1: *T*_(19)_ < 1, *p* = 0.41; 3: *T*_(19)_ = −2.20, *p* = 0.041; 4: *T*_(19)_ = −2.48, *p* = 0.023; 5: *T*_(19)_ = 1.41, *p* = 0.18]. Bonferroni-corrections regarding the post-hoc tests were applied for all conducted experiments.

#### Experiment 2

In parallel to Experiment 1, the percentage of correct responses showed a clear tendency to increase with an increasing size of the “pacman,” again for both presentation conditions (see Figure 2B). In parallel to Experiment 1, we conducted a repeated-measures ANOVA with the same factors and observed a significant interaction between these factors [*F*_(4, 15)_ = 27.85, *p* < 0.001] as well as a significant difference between the two positions for Sizes 1 and 3 [1: *T*_(18)_ = 5.59, *p* < 0.001; 3: *T*_(18)_ = 3.67, *p* = 0.002], but not for 2, 4, and 5 [2: *T*_(18)_ = 1.32, *p* = 0.21; 4: *T*_(18)_ = 1.76, *p* = 0.10; 5: *T*_(18)_ < 1, *p* = 0.61].

#### Experiment 3

Performance was higher, if the 3-2-3 array was presented in the Front and tended to increase with an increasing size of the “pacman” for both presentation conditions (see Figure 3A). The repeated measures ANOVA with Size and Position as independent factors revealed a significant main effect for Size [*F*_(4, 13)_ = 10.81, *p* < 0.001] and Position [*F*_(1, 16)_ = 9.81, *p* = 0.006] but no significant interaction [*F*_(4, 13)_ = 1.38, *p* = 0.30].

#### Experiment 4

Performance tended to increase with an increasing size of the “pacman” (Figure 3B). The repeated-measures ANOVA revealed a significant interaction between the two factors [*F*_(4, 13)_ = 4.58, *p* = 0.02]. By comparing Front and Back positions with paired *T*-tests for each size, we revealed significant differences for Size 2 [*T*_(15)_ = 3.34, *p* = 0.004], but not for Sizes 1, 3–5 [1: *T*_(15)_ < 1, *p* = 0.45; 3: *T*_(15)_ = −2.05, *p* = 0.60; 4: *T*_(15)_ < 1, *p* = 0.91; 5: *T*_(15)_ < 1, *p* = 0.48].

#### Experiment 5

Also in this experiment, performance showed a clear tendency to increase with an increasing size of the “pacman” (see Figure 3C). Two squares were perceived equally large if the square in the Front condition was 41% larger than in the Back. With the adjusted square, performance again increased with an increasing size of the “pacman” (see Figure 3C). For the main experiment, the repeated measures ANOVA revealed a significant main effect for Size [*F*_(4, 14)_ = 9.61, *p* = 0.001], but not for Position [*F*_(1, 17)_ = 2.97, *p* = 0.13]. The interaction effect was also not significant [*F*_(4, 14)_ < 1, *p* = 0.70].

#### Comparison experiment 1 vs. 3

To directly test for effects of local details, we performed a three-way ANOVA with Size (1–5), Position (Front, Back) and Experiment (1 and 3) as independent factors. This ANOVA revealed a significant three-way interaction of Size, Position and Experiment: [*F*_(4, 34)_ = 8.05, *p* < 0.001] that strengthens the assumption for a crucial role of the visual context regarding effects of visual constancy in visual integration.

## Discussion

The present study aimed to investigate the role of size constancy for global recognition in a task requiring the integration of local elements into a global object. We took advantage of the “Kanizsa” illusion while size constancy was achieved by a 3D-perspective in which the “Kanizsa” illusion was placed. According to previous observations emphasizing the length of the illusory contours as a crucial factor for the perception of “Kanizsa” triangles (Kojo et al., 1993; Liinasuo et al., 1997), we assumed that global recognition performance was lower if the local elements were perceived to be further away from each other (Beck, 1975). A general decrease in recognition performance was thus assumed for the Back compared to the Front. In line with these predictions is the data of Experiment 3, demonstrating lower global recognition performance of the target object in the Back than in the Front condition. Noteworthy, in this experiment only global information of the visual background was available, while local information (individual stones, texture) was removed. A similar but not significant pattern was observed in Experiment 5 after the size of the gray square was adjusted and thus equally large perceived in the Front and Back.

In this context, data from patients with simultanagnosia, a rare neurological disorder describing the inability to perceive a global Gestalt (Bálint, 1909), should be noted: global recognition performance in simultanagnosics can be modulated by the spatial distance between local elements and improves with smaller distances between elements (Huberle and Karnath, 2006). Further work indicated a key role of the visual angle together with the retinal image for global object recognition (Huberle et al., 2010). It could be demonstrated that rather the retinal image than the physical size of an object has a major impact on global perception. In addition, saliency has been linked to global recognition performance in simultanagnosics (Huberle and Karnath, 2010). However, the current results extent previous work and indicated that the perceived size might be more important for the recognition of a global Gestalt than its physical size. Further, the present findings cannot simply be attributed to retinal images and saliency, which were identical between the Front and Back condition. The explanation might rather be found in a systematic effect of size constancy on global perception that is attributed to the limited amount of local context information immediately available to the observer. In case of the absence of local context information a pronounced effect of size constancy on global perception is evident. Size constancy underlies the observation that separate objects presented in an enriched visual context are rather perceived at a subjective size. A recent study investigating size constancy in a virtual reality environment demonstrated that despite of an identical physical and retinal image perceived object size was mainly determined by perspective manipulations (Kenyon et al., 2008).

The results of the remaining experiments indicated a more complex interaction between size constancy and the integration of local elements into a global object. First, the data of Experiment 1 suggested an influence of local context information, namely the stone-like surface used for the wall. If the “pacmen” were large, the results were similar to Experiments 3 and 5. However, for small sizes of the “pacmen” the reversed pattern—higher global recognition performance in the Back than in the Front—was observed. Similar results became evident for Experiment 4. The differences in the global recognition performance between Experiments 4 and 5 might also be explained by local context information. In Experiment 5, the gray square in the Front condition was more than 40% larger than in Experiment 4 and therefore covered more of the local surround. Moreover, the stimulus presentation of Experiment 3 is comparable to earlier work by Beck (1975), where global stimuli were also presented on a plane background. Further, local context information should also be regarded together with visual crowding, the interaction between nearby contours or “visual clutter” on visual discrimination and object recognition (Levi, 2008; Pelli and Tillman, 2008). “Visual clutter” surround global targets might disturb global recognition performance (Dakin and Baruch, 2009; Kingdom and Prins, 2009; Lau and Cheung, 2012; Robol et al., 2012). The idea of an interaction with local context information was further strengthened by the results of Experiment 2. Local context information with a minimized global surround (flat wall with small and large stones) showed a lower global recognition performance if large stones (equal to the Front) were used instead of smaller ones (similar to the Back). Moreover, the results of this experiment suggest that global recognition performance in the remaining experiments was mainly influenced by perspective manipulations and was independent from local context information. However, the present data cannot answer the question if local details changed the perceived size between local elements or influenced visual integration in general. Evidence about visual crowding and object perception (Levi, 2008; Pelli and Tillman, 2008), nevertheless, suggests a general influence of local details on integration processes. These observations reported here were mainly restricted to Sizes 2–4 while differences between Back and Front presentation for Sizes 1 and 5 were less pronounced over all experiments. We address this effect to a higher experimental sensitivity for the middle sizes of the stimulus spectrum. Moreover, missing variability for Size 5 can be attributed to a general ceiling effect for the ‘easiest’ condition, while Sizes 2–4 appeared to be more sensitive to reveal perspective differences between the Back and Front presentation.

Recent work observed a faster recognition of meaningful arrays creating illusory contours compared to random configurations of local elements in a noisy visual background indicating also an involvement of early visual areas in integration mechanisms (Wang et al., 2012). Further, neuroimaging studies have identified distinct neuronal networks of illusory contour processing (Hirsch et al., 1995; Ffytche and Zeki, 1996; Halgren et al., 2003; Maertens and Pollmann, 2005, 2007). However, the evidence of influences of size constancy on Gestalt perception supports the view of contributions of later visual areas to processes of visual integration. Various studies showed that functions of object perception get affected by size constancy (Emmert, 1881; Fitzpatrick et al., 1982; Kenyon et al., 2008) and localized object processing beyond early visual areas along the ventral visual pathway (Ungerleider and Mishkin, 1982; Goodale and Milner, 1992; Grill-Spector, 2003). Therefore, the presented results demonstrating influences of size constancy on Gestalt perception are in line with various findings from patients with simultanagnosia and healthy subjects attributing global perception to posterior parietal brain areas (Rizzo and Hurtig, 1987; Friedman-Hill et al., 1995; Rafal, 1997; Karnath et al., 2000; Tang-Wai et al., 2004; Valenza et al., 2004; Himmelbach et al., 2009; Huberle and Karnath, 2012; Thomas et al., 2012).

The current results suggest an important role of size constancy on global perception of hierarchically organized visual stimuli. In accordance with previous findings, the distance between local elements in illusory global objects composed of local elements appears to be a crucial factor for global recognition. Emphasizing the importance of the perceived size of a global figure in visual integration, the data extend previous findings in patients with simultanagnosia that attributed global recognition performance to the retinal size rather than the physical. To our knowledge, the work shown here represents the first manipulation of global recognition with mechanisms of size constancy preserving a constant retinal image and, thus, highlights the role of the perceived size of a global object in the process of visual integration. Size constancy influences our perception and leads to an enlarged inter-element spacing in hierarchically organized global figures. Finally, local context information shows an interaction with size constancy.

### Conflict of interest statement

The authors declare that the research was conducted in the absence of any commercial or financial relationships that could be construed as a potential conflict of interest.
